# Self-ingested intraduodenal foreign bodies – expectancy or surgical sanction?

**Published:** 2014-09-25

**Authors:** S Petrea, I Brezean

**Affiliations:** * “I. Juvara" Surgery Clinic, “Dr. I. Cantacuzino" Hospital, Bucharest

**Keywords:** self-harm, ingestion, foreign bodies

## Abstract

Abstract

Self-harm is a frequent pathology amongst psychiatric patients and in the penitentiary environment. Multiple self-aggression types are described, but, by far, the practice most frequently met inside the Romanian penitentiary environment is foreign body (FB) ingestion. The paper aims to show aspects pertaining to the presence of intraduodenal foreign bodies, both in simple cases and in cases that ended with a perforation, using a number of 47 cases registered between 2003 and 2010 in “Rahova" Penitentiary Hospital. The paper also focused on particular aspects linked to intraduodenal foreign body surgical accessibility.

Abbreviations: FB=foreign bodies; OR=operating room; GEA=gastroenteroanastomosis

## Introduction

Self-aggression is generally a non-fatal act, through which a person deliberately provokes a lesion or ingests a substance in excess of any known therapeutic dose or medical prescription. The act is clearly non-accidental, but with no clear indication of the presence or absence of a death wish [**1**]. This type of pathology is present in psychiatric patients and is frequently seen amongst penitentiary inmates, but rarely discussed in medical literature – often, as case presentations.

Psychiatric examination notes as causes of self-harm: psychiatric pathology, a wish to obtain better detention conditions or a desire for other benefits. They can also constitute forms of protest against carceral injustice or forms of manifesting one’s freedom to act upon one’s body as one sees fit.

762 cases of self-aggression inside the surgical service of the “Rahova" Penitentiary Hospital were studied, all admitted and treated in a 7-years interval (2003- 2010). 472 cases recognized foreign body ingestion as the mechanism of self-aggression. 45 cases developed perforative complications, with a different impact upon the patient’s status. In 10% of the cases, the foreign body was impacted at the duodenal level. While the general frequency of complications is around 10% for the majority of the ingested FBs, in the case of intraduodenal FBs, the complication frequency rises to 34% (three times higher).

Intraduodenal foreign body ingestion cases accompanied or not by complications, will be discussed in the present study.

## Material and method 

A retrospective study of 47 cases of voluntarily ingested intraduodenal FBs admitted and treated in “Rahova" Penitentiary Hospital between August 2003 and February 2010 was attempted.

Self-aggressive patients had a typed follow-up chart including the following data:

1.general patient data; 2. self-harm history, surgical intervention history; 3. data about the FB; 4. time elapsed between the moment of self-aggression and the moment of surgical intervention; 5. clinical diagnosis; 6. laboratory/paraclinical diagnosis; 7. lesion topography; 8. surgical intervention and lesion complications; 9. post-operative evolution.

The analysis of intraduodenal FBs based on these data is presented below.

## Results 

1. Age, sex: age-group sample allotment showed a concentration of cases in the third and fourth age decades (25 patients between the ages of 21-30 and 14 patients in the 31-40 age groups); over ¾ of patients belonged to the aforementioned decades. All the patients were male.

2. FB ingestion history: most of the patients were at the first episode (20 cases), a quantifiable percentage (49%) had a history of two to four self-aggression episodes, while cases with more attempts were singular. The extreme is represented by a patient with 27 self-aggression episodes.

3. Data about the FB

The studied cases totaled a number of 81 FBs. 26 cases of single-body ingestion and 13 cases of double ingestions were found; the rest of the patients habitually ingested between 3 and 5 FBs.

All the FBs ingested were metallic, such as: cutlery handles (47), wires (11), needles (11), whole pieces of cutlery or sizable fragments (3 forks, one teaspoon and a knife blade), 2 steel reinforcing bars, one each of ribbon iron, iron file rail, construction nail, ballpoint pen, rolled-up tin can lid.

The FBs’ dimensions were small (0-5 cm) in 7 cases, medium (5-10 cm) in 19 cases, large (8-15 cm) in 47 cases, very large (over 15 cm) in 8 cases. An analysis of the edge and margin structure showed that a number of 25 FBs were sharp-edged, 5 were blunt-edged, 49 had cutting edges and two cases had both cutting and sharp edges.

4. Time interval between self-aggression and medical examination

The delay was generally due to the patients, either hiding the self-aggression or acknowledging it but refusing medical attention as they hoped for a subsequent complicated evolution of their lesions and tried to use this as a lever in blackmailing the penitentiary staff and obtaining different advantages. On the other hand, some delays might have been due to organizational deficiencies inside the penitentiary system (limited transportation towards the penitentiary hospital) as well as deficient teamwork between penitentiaries and the local hospitals in their proximity [**2**].

Only 18 patients were admitted to our hospital in the first week after the self-aggression moment; 38 patients were admitted in the first month, while the rest were admitted in a time interval ranging from one month to one year. The high mean duration of time between self-harm and hospital admission (32,4 days) can explain the low rate of success for endoscopic extractions and the high incidence of complications. For perforating complicated intraduodenal FBs the mean time duration between self-aggression and hospital admission was 44,5 days.

5. Clinical diagnosis

The clinical symptomatology was varied, the patient could be asymptomatic or could present with intense algic complaints. There was a link between the absence or presence of complications and symptomatology. Thus, of the 16 asymptomatic cases, only one had a complication, while symptomatic cases equally belonged to patients with and without complications.

Pain was the most frequently encountered sign (31 cases) with a superior abdominal distribution; two cases associated peritoneal irritation signs, due to a free perforation of the superior duodenal knee with localized peritonitis in the first case, and to a blocked perforation of the inferior duodenal knee with secondary septic hepatic dissemination in the second. In the septic dissemination case, the patient associated high fever and a general deterioration of the habitus. Major complications, such as peritonitis or sepsis, alter the general clinical pattern in relevant ways, allowing a quick diagnosis of complications.

6. Paraclinical diagnosis 

Biological diagnosis: most patients had no biological alterations. We found variation of the biological constants in just 4 cases. Isolated leukocytosis was encountered in two cases, liver cytolysis tests were increased in one patient with DII perforation associated with liver cirrhosis. The case with blocked DII perforation and secondary septic liver disseminations also had a marked anemic syndrome and hepato-renal failure.

Image diagnosis

Abdominal X-ray

It was a useful diagnostic tool, as it pinpointed the radio-opaque image and showed the FBs’ physical characteristics (number of FBs, size, edge and extremity aspect). The interventional moment can usually be assessed by using this data.

**Fig. 1:**
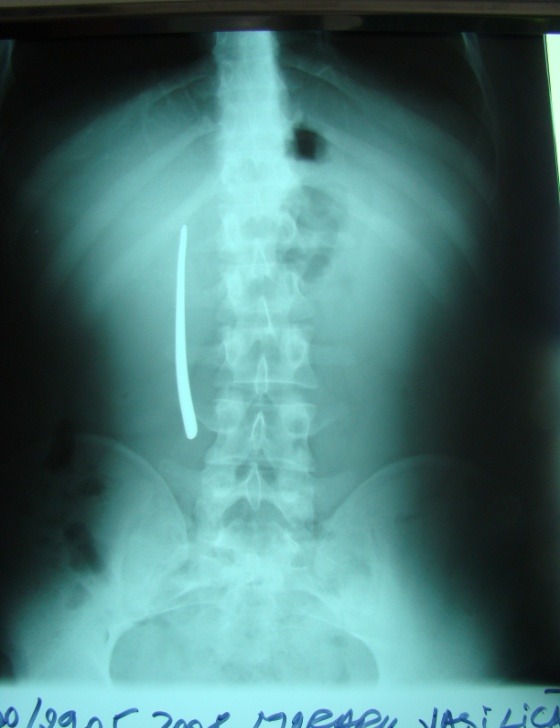
FB (cutlery handle) in the DII

The position of the radio-opaque image generally corresponded to the anatomic projection of different duodenal segments upon the abdominal wall; one noted the right-edge superposition of the FB image upon the lumbar spine, characteristic of DII FB positioning – an image we encountered in 19 (68%) of the 28 cases presenting with DII-placed FBs (**Fig. 1**).

 In one case of duodeno-hepatic penetration, one hydro-aerial level was present at the superior edge of the FB.

Contrast medium X-ray (using Gastrografin)

**Fig. 2:**
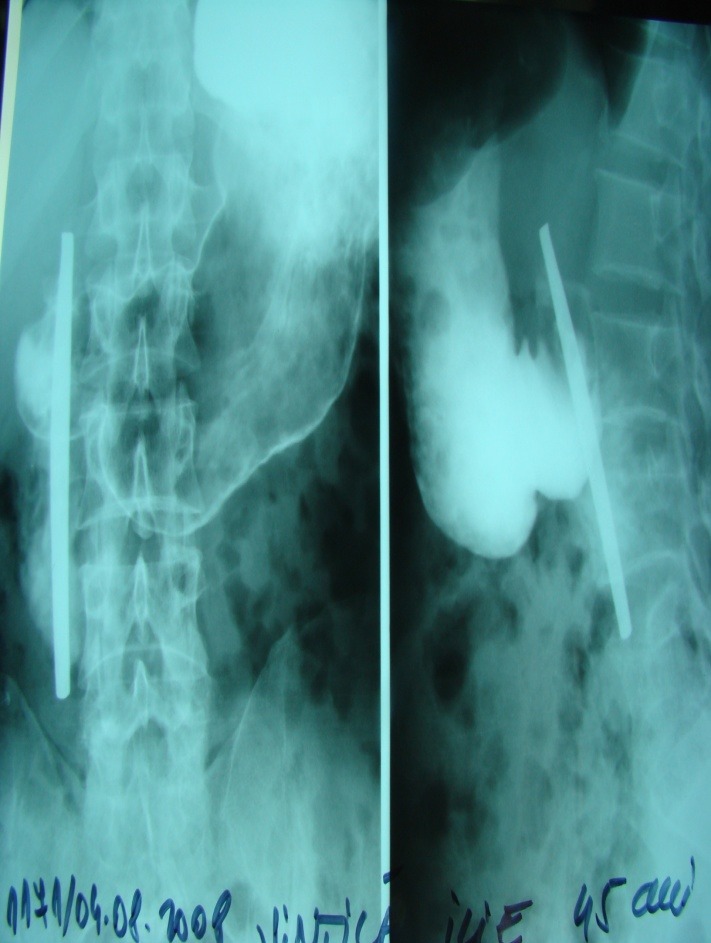
FB (cutlery handle) in the DII, contrast-medium X-ray (front, profile) using Gastrografin

Can be used when the patient does not accept endoscopic procedures, in order to ensure that the FB is placed inside the duodenum (**Fig. 2**).

Superior digestive endoscopy

Represents both a diagnostic and a therapeutic method, and was performed in 11 out of the 47 investigated patients.

The rest of the patients could not benefit from this procedure for various reasons, amongst which we noted:

- physical characteristics of the FB (over 20 cm long wires with bent and rounded ends – 2 cases; large dimensions and rounded ends with difficulties in obtaining a purchase point for the surgical forceps in the case of an 18 cm long ballpoint pen ingested together with a cutlery handle; extreme weight of the FB in the case of 2 large pieces of reinforcing bar). 

- the FB had already passed the DII – diagnosed by X-ray (2 cases).

- the patient’s refusal to submit to investigations (2 cases).

Often, the cause was the absence of a qualified endoscopic specialist (28 cases).

This maneuver demanded consistent human and material resources, including special grasping of surgical forceps of different sizes, overtubes for gastro-esophageal junction passage, etc.

Abdominal ecography and/or tomography

Can be used for the FBs positioned in the DII, whenever a suspicion of lesion complication exists, for assessing the existence of hepatic penetration, in the case of fever syndrome associated to FB ingestion, or for the identification of septic metastases or pathological liquid presence inside the peritoneal cavity.

7. FB topography

We noted duodenal FBs as unique localization, duodenal FBs associated to other localizations and FBs occupying different successive digestive segments – stomach, duodenum.

The solitary localization was noted in 36 cases – 33 FBs inside the DII, 2 in the DIII and one in the DIV segments. 10 cases of intraduodenal FBs, associated FBs with different localizations, such as intragastric in 8 cases and intracolic in 2 cases. One case in which the FB was over 15 cm in length covered two successive digestive segments - stomach and duodenum.

8. Evolutive complications of intraduodenal positioning of FBs

Are frequently seen in this type of FB positioning, in the paper - 16 of the 47 cases we studied (34%). Perforative complications were frequently noted in the DII localization, represented by duodeno-hepatic penetration (9 cases), and a case of: duodenal fistula with localized peritonitis; double blocked penetration of the superior and inferior duodenal knees; triple penetration of the superior duodenal knee, inferior duodenal knee and duodenal sulcus; blocked perforation of the inferior duodenal knee.

The other 3 complications were noted in the case of FBs positioned inside the DI (one case of duodeno-hepatic penetration) and the DIII (two blocked perforations, one with septic hepatic determinations). 

The mean time between the self-aggression and hospital admission in these cases was 44,5 days, and 48 days until the surgical sanction. 

Complications occurred in 2 of the operated cases in the first 10 days after self-aggression, in 6 cases operated in the 11-20 days interval and in 8 cases operated between 21 and 30 days. The 29 FBs that caused the aforementioned complications were solitary in half the cases, 2 FBs in 5 cases, 3 or 5 FBs in other 3 cases. Most complications (81%) were caused by FBs more than 10 cm long (11 lesions caused by large FBs and 2 lesions caused by very large FBs), with cutting edges (8 cases) or with sharp edges (6 cases).

Both the duodenum and the rectum are parts of the digestive system where complications caused by cutting FBs are more frequent than the ones caused by sharp FBs [**2**].

This can be explained by the fact that most of the FBs present at the duodenal level are cutlery handles, which constantly measure over 10 cms in length and are relatively heavy. Once impacted inside the inferior duodenal knee, these provoke lesions through erosion of the duodenal wall caused by the cutting edge of the fragmented cutlery and also because of duodenal wall extension due to the FBs dimensions.

9. Duodenal FB treatment

Endoscopy is indicated as treatment for the FBs positioned in the first duodenal segments, for large FBs occupying successive digestive segments or when an intraduodenal positioning is associated with other positioning. Taking into account the time lapse between self-aggression and hospital admission , the frequency of complications – especially for DII-placed FBs and their often large dimensions, the maneuver is often difficult, because of a penetration of the superior or inferior duodenal knee - or both - with subsequent FB inclavation, which makes any extraction method impossible or accompanied by a bleeding upon attempting to mobilize them. There is no contraindication for this therapeutic maneuver in the case of adjacent organ penetration or blocked perforation – but it is forbidden when signs of acute abdominal syndrome caused by perforation are present.

In the case of complicated FBs with adjacent organ penetration, multiple FBs or an incompliant patient, an extractive maneuver under general anesthesia, with OTI in the OR, can be considered – allowing the unsuccessful maneuver to be continued by a classical surgical intervention. Obviously, in the case of a duodenal FB complicated by penetration or blocked perforation there is no chance of natural elimination, as is also the case for FBs which stay in the duodenum for more than 2 weeks after self-aggression.

Endoscopy was successful in 6 cases, in 4 of them solitary FBs were extracted; in one case two, respectively three FBs were extracted. All the extracted FBs were positioned inside the DII and caused no complications and were represented by cutlery handles in 5 cases and needles in one case. 

General intravenous anesthesia was used in 5 of the 6 cases; midazolam, atropine, pethidine, H2-blockers were used.

**Fig. 3:**
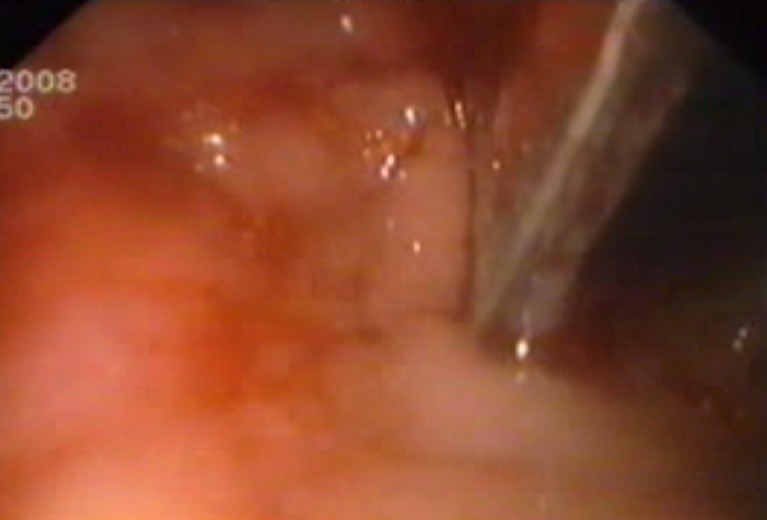
Intraduodenal FB (cutlery handle) with superior duodenal knee fistula

The maneuver was unsuccessful in 5 cases, either due to FB physical properties (number, dimensions, weight) or to their distal positioning (DIII) with difficult access, or even to bleeding complications caused by FB mobilization and subsequent penetration of another organ; last but not least, the endoscopist’s lack of experience and manuality, in the case of a 10cm long cutlery handle (**Fig. 3**).

Surgical intervention 

The primary choice in the case of acute abdominal syndrome signs and symptoms is associated to the presence of an intraduodenal FB. Other indications are: intraduodenal FB at more than 10 days after the aggression, failure of endoscopic extraction or endoscopic evidence of complications, FBs with certain physical characteristics (over 15 cm in length and with sharp edges) [3,4].

In order to avoid the mobilizing of duodenal segments and pancreaticoduodenal education, the lesion can be accessed through segments immediately adjacent to the lesion and FB position, such as the stomach or first jejunal segment, leaving the duodenal lesion in situ [**2**]. In the case of DII, the FBs with superior or inferior penetration, access can be gained through the non-interested lesion extremity. If this is not possible or if the fistula accidentally opens, FB extraction, lesion edge reshaping and duodenorrhaphy can be attempted. In multiple FB, positioning inside successive digestive segments access can be obtained through a single incision on the mobile segment (gastrostomy for gastric and duodenal positioning of the FB).

In the case of FBs positioned in different segments, multiple incisions are needed, after trying to group the FBs in an attempt to minimize the number of needed incisions.

One last case - a solitary FB (14-cm long cutlery handle) was discharged on demand and lost from subsequent surgical evidences. 

**Table 1** shows the types of lesion and their mode of treatment.

**Table 1:**
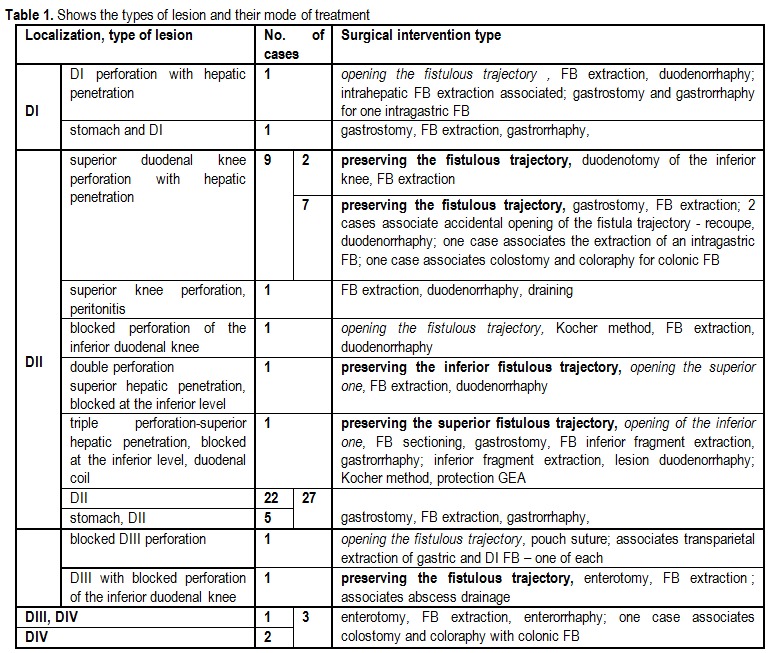
Shows the types of lesion and their mode of treatment

10. Intraoperative complications

We noted one complication, an enteral continuity solution in a multiply operated surgical patient (24 operations) in which an enterorrhaphy was needed.

11. Postoperative evolution

The 40 surgical interventions presented with 4 complications and one death.

Parietal complications were represented by one case of free - self-induced evisceration on the third postoperative day, where we opted for a total plan abdominal wall suture.

Abdominal complications were represented by one case of dynamic ileus in a patient with complicated surgical history – which was solved by medical treatment.

General complications were represented by liver cirrhosis decompensation and transitory cerebral ischemic accident – one case each.

The fatal evolution was noted in a patient presenting with blocked DII perforation and liver abscesses, who was admitted 4 months after the self-aggression act, with severe sepsis, and who developed acute bilateral broncho-pneumonia and ARDS shortly after surgery.

## Discussions

Taking into account the lack of literature data when dealing with self-aggression through FB ingestion, discussions will be confined almost exclusively to analyzing the data from the present study.

Self-aggression through FB ingestion is a particularly pathology, usually described in prison inmate populations and psychiatric patients [**2**]. Existing data refer to accidental FB ingestion and are generally confined to case presentations. We found one other paper studying FB ingestions, that presents 542 cases of both involuntary and deliberate FB ingestions [**5**].

Age and sex patient allotment is represented by a predominance of third and fourth age decades and male sex amongst penitentiary population.

The ingested FBs are often solitary (55%), metallic, of large and very large dimensions (69%), cutting (60%) and generally represented by cutlery handles. 

Patient presentation for a medical examination is reduced in the first 7 days (38%), half of the patients being examined in the 7-30-days interval – in contrast to the mean time of 12 hours which a British study found in voluntary and non-voluntary FB ingestion amongst a number of 38 patients [**6**]. This obviously leads to an increase in the number of complications and surgical interventions.

The clinical examination is important for the management of FB ingestion in order to note the onset of complications; the examiner must be alerted to the possibility of patient simulation, often encountered in our cases. With the exception of peritoneal irritation cases, symptomatology is generally minimal or absent.

Although literature data considers a length of 6 cm [7-9] and a diameter of 2 cm as limiting pyloric effraction, our data show that most of the intraduodenally-captive FBs are large and very large ( 55 FBs over 10 cm vs. 26 FBs under 10 cms); with a prevalence of cutting FBs (49) over sharp FBs (25).

X-ray examination plays the main role both in the diagnosis and choice of operative moment – either by showing certain FB characteristics, or by noting images suggesting complications (rare in our case), or even by projecting the FB in the same place over a period of time, an aspect inductive of fistulas. The persistence of the FB in the DII for more than 10 days after self-aggression usually indicates the presence of a fistula [**2**], although this was also found in 2 cases which we operated earlier than 10 days after the ingestion. Blocked duodenal perforation, sometimes asymptomatic, can be diagnosed by echography (FB edge positioned outside the digestive walls, frequently intra-hepatic – or the presence of a FB, identified by X-ray, completely inside the liver).

Therapeutic endoscopy is a prime intention maneuver in the case of intraduodenal FBs. In order to be efficient, it needs a set of simultaneous conditions that our study could not always offer - first, human and technically appropriate expertise. The other conditions are the following: patient acceptance, physical characteristics, lesion particularities and positioning types of the FB (reasonable weight, dimensions and number of FBs allowing manipulation, an absence of perforative complications, FB positioning above the inferior duodenal knee). We cannot quantify the “reasonable" concept when dealing with a low number of endoscopic extractions. In the majority of papers studied, small patient samples were used and a low extraction rate was practiced (10-20% of small dimension, low weight FBs) [5,7].

It is accepted that endoscopic extraction should be attempted as soon as possible for sharp, cutting, or over-10-cm long FBs – but the condition is incumbent upon the time of addressing medical care, very long in our study when compared to the 6-hours mean time for the performance of the maneuver noted on a lot of 414 FB ingestions (often alimentary) in a British study [**10**].

Literature data generally indicate a complication level of under 1% [3,4,7,11-15] but there is one study with a rate of 5-7% [**16**]. Although the duodenum is often a FB impaction zone, it is not recognized as a complication site [4,7,11-14,17-18]. Lesions were of a complicated type in 34% of our cases, most of them being blocked perforations or penetrative type lesions; we noted a single case of free perforation in a patient with associated hepatic pathology. This contradicts a study that found a complication rate of 65% for perforations accompanied by peritonitis in a lot of 33 patients [**13**].

From a surgical point of view we noted the possibility and usefulness of indirect access to intraduodenal FBs, even for cases already complicated by penetration, by using mobile superimposed superior digestive segments (stomach) or inferior ones (first jejunal coil) [**2**]. 

Postoperative complications have no specific character, and are generally unrelated to the surgical act per se.

Death of a multiply-operated patient (27 surgical interventions, all for the same type of pathology) who presented 4 months after FB ingestion, in severe sepsis, with liver abscesses – due to repeatedly refusing the operation - is, we believe, the only case of voluntary FB ingestion with blocked perforation and secondary hepatic abscesses present in literature to this date.

## Conclusion 

Self-aggression through FB ingestion is a frequent pathology inside Romanian penitentiaries.

Duodenal impacted FBs are often large and very large, cutting or sharp (aspects dictated by the type of FB frequently found at this level), presenting with no specific symptomatology.

Abdominal X-ray is the elective diagnostic method, useful for the choice of the interventional moment too, depending on the physical characteristics of the FB. Routine echographies can objectify DII penetration in the case of intraduodenal FBs. 

Complications frequently occur in the intraduodenally-positioned FBs (1/3 of cases); the longer the time interval between the time of ingestion and the starting of the treatment, the higher the complication rate.

Digestive endoscopy extraction should be tried in all uncomplicated cases, but it needs human and technical resources.

Surgical treatment is the main therapeutic method for intraduodenal-positioned FBs; duodenal access by using adjacent digestive segments is to be preferred in all cases, with the exception of free perforations, its morbidity being practically null.

The choice of the operative moment is dictated by the physical characteristics of the FB, the presence or absence of acute abdominal syndrome, or FB remanence inside the same digestive segment for more than 10 days after the moment of ingestion.

An improvement in penitentiary life conditions, better communication between staff and inmates, instruction, education, learning, new healthy and civilized ways of spending free time, respect for the inmates’ civil rights according to the law are modalities which could lead, in time, to a decrease of FB ingestion cases [**2**].
